# Supplementary material for: First-line antiretroviral therapy initiation for newly diagnosed people with HIV in the Netherlands: A retrospective analysis from 2016 to 2020
Source: PLoS One. 2024 Jul 26;19(7):e0307963. doi: 10.1371/journal.pone.0307963 (PMC11280218; doi:10.1371/journal.pone.0307963)
Supplement: S1 Table — (DOCX) [file pone.0307963.s001.docx]

**Supporting Information**

S1 Table: Distribution of STR versus branded MTR vs generic MTR from 2016 – 2020.

Table. Distribution of STR versus branded MTR vs generic MTR from 2016 - 2020

| Center | 2018 STR vs bMTR vs gMTR (%) | 2019 STR vs bMTR vs gMTR (%) | 2020 STR vs bMTR vs gMTR (%) | |
| --- | --- | --- | --- | --- |
| 1 | 79 vs 5 vs 16 | 37 vs 1 vs 62 | 9 vs 0 vs 91 | |
| 2 | 65 vs 0 vs 35 | 44 vs 4 vs 53 | 37 vs 9 vs 54 | |
| 3 | 67 vs 22 vs 11 | 97 vs 0 vs 3 | 95 vs 0 vs 5 | |
| 4 | 83 vs 8 vs 9 | 82 vs 3 vs 15 | 33 vs 0 vs 67 | |
| 5 | 89 vs 11 vs 0 | 100 vs 0 vs 0 | 92 vs 0 vs 8 | |
| 6 | 96 vs 4 vs 0 | 89 vs 0 vs 11 | 100 vs 0 vs 0 | |
| 7 | 65 vs 32 vs 3 | 83 vs 0 vs 17 | 95 vs 0 vs 5 | |
| 8 | 65 vs 6 vs 29 | 65 vs 4 vs 30 | 22 vs 0 vs 78 | |
| 9 | 85 vs 8 vs 8 | 92 vs 4 vs 4 | 100 vs 0 vs 0 | |
| 10 | 67 vs 10 vs 24 | 75 vs 0 vs 25 | 40 vs 0 vs 60 | |
| 11 | 92 vs 4 vs 4 | 95 vs 5 vs 0 | 100 vs 0 vs 0 | |
| 12 | 82 vs 0 vs 18 | 100 vs 0 vs 0 | 75 vs 0 vs 25 | |
| 13 | 0 vs 8 vs 92 | 0 vs 6 vs 94 | 23 vs 0 vs 77 | |
| 14 | 94 vs 6 vs 0 | 95 vs 5 vs 0 | 89 vs 0 vs 11 | |
| 15 | 50 vs 42 vs 8 | 75 vs 25 vs 0 | 63 vs 38 vs 0 | |
| 16 | 100 vs 0 vs 0 | 100 vs 0 vs 0 | 100 vs 0 vs 0 | |
| 17 | 100 vs 0 vs 0 | 63 vs 0 vs 38 | 100 vs 0 vs 0 | |
| 18 | 76 vs 6 vs 18 | 100 vs 0 vs 0 | 90 vs 0 vs 10 | |
| 19 | 78 vs 0 vs 22 | 75 vs 0 vs 25 | 33 vs 0 vs 67 | |
| 20 | 100 vs 0 vs 0 | 88 vs 0 vs 13 | 11 vs 0 vs 89 | |
| 21 | 92 vs 0 vs 8 | 100 vs 0 vs 0 | 100 vs 0 vs 0 | |
| 22 | 88 vs 0 vs 13 | 33 vs 33 vs 33 | 33 vs 0 vs 67 | |
| 23 | 100 vs 0 vs 0 | 100 vs 0 vs 0 | 100 vs 0 vs 0 | |
| 24 | 40 vs 20 vs 40 | 71 vs 0 vs 29 | 25 vs 0 vs 75 | |
| 25 | 50 vs 0 vs 50 | 67 vs 0 vs 33 | 67 vs 0 vs 33 | |
| 26† | 100 vs 0 vs 0 | - | - | |
| 27† | 67 vs 0 vs 33 | 100 vs 0 vs 0 | - | |
|  |  |  | |  |

† during 2019 and 2020, the two centers (Centers 26 and 27) were closed and were no longer functioning as HIV treatment centers
